# Fabrication of High-Performance Asphalt Mixture Using Waterborne Epoxy-Acrylate Resin Modified Emulsified Asphalt (WEREA)

**DOI:** 10.3390/polym16192743

**Published:** 2024-09-27

**Authors:** Dongwei Chen, Hao Wu, Xiaobao Chen, Yiqun Zhan, Surajo Abubakar Wada

**Affiliations:** 1 School of Civil Engineering, Central South University, 22 South Shaoshan Rd., Changsha 410075, China; chendw1717@mails.jlu.edu.cn (D.C.); 15594532771@163.com (X.C.); zhanyiqun@csu.edu.cn (Y.Z.); srjwada@gmail.com (S.A.W.); 2 Department of Civil Engineering, Ahmadu Bello University, Zaria 810107, Nigeria

**Keywords:** waterborne epoxy-acrylate resin emulsified asphalt (WEREA), cold mix asphalt (CMA), rheological properties, fatigue resistance, mix design method, pavement performances, high-temperature performance

## Abstract

Existing research shows that using waterborne epoxy resin (WER) instead of emulsified asphalt as the binder for cold mix asphalt (CMA) can enhance the rutting resistance, high-temperature performance, fracture performance, and early performance of CMA. In order to eliminate the potential drawbacks such as insufficient strength and low-temperature performance of CMA during application, a novel method was proposed in this study for the preparation of waterborne epoxy-acrylate resin (WER), specifically tailored to modify emulsified asphalt, resulting in waterborne epoxy-acrylate resin emulsified asphalt (WEREA). The modification effect of WER on emulsified asphalt was evaluated through rheological tests and direct tensile tests. A modified design method based on the conventional Marshall design method was proposed to determine the optimal mix proportions, including the key parameters of specimen compaction and curing. The results revealed that the incorporation of WER led to a substantial improvement in the complex shear modulus and a concurrent decrease in the phase angle. When the temperature exceeded 60 °C, the phase angle exhibited a diminishing trend, indicative of a reduced viscosity as temperatures escalated. As the WER content increased, a decrease in the direct tensile strain rate was observed, accompanied by a substantial elevation in direct tensile strength. At various stress levels, the shear strain of WEREA decreases with increased content of WER, indicating that the incorporation of WER can enhance the hardness of emulsified asphalt and improve its deformation resistance. The results from MSCR tests indicate that WER could significantly improve the elasticity and hardness of emulsified asphalt, transitioning it from a viscoelastic material to an elastic material, thereby improving its deformation resistance, resistance to rutting, and high-temperature performance. The results of fatigue life are consistent with those of the amplitude sweep, both reflecting the improvement of resistance to deformation of emulsified asphalt by WER. This indicates that WER has a significant improving effect on the fatigue resistance of emulsified asphalt. Furthermore, the Marshall design tests further confirmed the advantages of WEREA in asphalt mixtures. The optimal preparation for the WEREA mixture was proposed as follows: double-sided compaction for 50 times each, aging at 60 °C for 48 h, optimal moisture content of 5.14%, cement content of 2.5%, and emulsion content of 8.4%. The optimal mix proportions identified through these tests yielded asphalt mixtures with significantly improved stability, reduced flow value, and enhanced rutting resistance compared to the hot-mix asphalt mixture (HMA) of AC-16. These findings suggest that WEREA has the potential to significantly enhance the durability and longevity of asphalt pavements. For future applications, it can be explored for use in producing cold recycled asphalt mixtures. In addition to designing the WEREA mixture according to AC-16 gradation, consideration can also be given to using a gradation with a smaller nominal maximum aggregate size for the application in the surface layer or ultra-thin wearing course.

## 1. Introduction

Road construction is essential for urban growth and transportation development. Traditional hot mix asphalt (HMA), widely used in pavements, offers great performance with benefits like high strength, durability, and ease of construction, which align with urban transportation needs. Nonetheless, its use can also result in harmful gas or smoke emissions [1,2,3]. The emission of volatile harmful substances can potentially threaten both the environment and human health, including irritating gases like H_2_S, SO_2_, and NH_3_. As the transportation industry progresses, there is a growing need for environmentally friendly and sustainable practices in highway construction and usage [4,5]. Finding an eco-friendly alternative material has become critical to meet national policy requirements and societal development needs.

In recent decades, cold mix asphalt (CMA) has gained more attention due to its lower mixing and construction temperatures (0~40 °C) [6]. While producing HMA requires 9 L of fuel and 8 kW of electricity per ton and emits 28.8 kg of CO_2_ per ton [7], CMA mixtures consume less fuel [8,9]. Consequently, CMA mixtures are better suited to current energy-saving and emission-reduction standards for asphalt mixtures. However, CMA uses emulsified asphalt as a binder, which has limitations such as lower early mechanical strength [10] and poor water resistance [11,12,13]. Moreover, the lower temperatures during CMA production result in less effective curing, and rutting is a frequent problem [14,15,16]. As a result, CMA does not meet the standards required for high-grade road structural layers, and CMA mixtures are usually utilized as preventive maintenance materials for roads [17,18,19]. Hence, it is essential to create CMA mixtures that are appropriate for high-grade road structural layers.

To improve the high-temperature performance and mechanical properties of emulsified asphalt, various additives, including styrene–butadiene–styrene (SBS) [20,21], styrene–butadiene rubber (SBR) [22,23], polyvinyl acetate (PVA) [24], and others are incorporated for modification. In addition to the modifiers mentioned above, as a polymer material, epoxy resin possesses many excellent properties, such as excellent adhesion, outstanding electrical insulation, and high mechanical strength. These characteristics make epoxy resin widely used in various fields, including construction, electronics, automotive, and aerospace. Choi et al. [25] studied the effects of moisture and heat on carbon fiber/epoxy composites used in aircraft. The moisture absorption and physical changes of the composites were measured, and it was found that the glass transition temperature decreased linearly with increasing moisture content. Konstantinova et al. [26] used 4-(β-carboxyethenyl) phenoxy-phenoxycyclotriphosphazenes (CPPP) as a curing agent and found that it can improve the adhesion of epoxy resin to steel and aluminum while also exhibiting high fire resistance, thermal stability, heat resistance, and resistance to fresh and saltwater, as well as low water absorption.

In view of the many excellent features it possesses, epoxy resin is frequently utilized as a modifier to greatly boost the performance of asphalt or emulsified asphalt. Adding epoxy resin to emulsified asphalt can significantly enhance its high-temperature performance and mechanical properties [27,28]. It effectively mitigates the issues of poor mechanical properties found in conventional asphalt emulsions. Unlike oil-based epoxy resins, waterborne epoxy resin (WER) is free of volatile organic compounds (VOC), which simplifies its storage and transportation. When WER is blended with asphalt emulsion, both the small asphalt particles and WER particles are uniformly dispersed within the suspension. As WER transitions from liquid to solid with the addition of other additives, the epoxy groups in the resin react with amino groups in the curing agent through dehydration condensation, leading to cross-linking and the formation of a large interpenetrating polymer network (IPN) [29,30,31]. Typically, an increase in WER content enhances the high-temperature resistance of the emulsified asphalt, though it may reduce its deformability.

In recent years, numerous researchers have been concentrating on CMA mixtures. Xiao et al. [32] investigated the effect of cement on CMA mixtures. The findings show that incorporating cement greatly improves the high-temperature stability and water sensitivity of cement-emulsified asphalt mixtures. Xu et al. [33] examined a new kind of SBS-modified emulsified asphalt mixture, which demonstrates superior mechanical performance and significant water stability. Liu et al. [34] found that compared to emulsified asphalt mixtures, the waterborne epoxy resin/styrene–butadiene rubber latex-modified emulsified asphalt (WSEA) mixtures exhibited improved rutting resistance, water stability, and interlayer shear strength. Xu et al. [35] investigated how particle size distribution impacts the engineering performance of CMA. The results reveal that the high-temperature stability of cement-emulsified asphalt is influenced by both aggregate gradation and adhesion properties. Yao et al. [36] found that the reinforcement and toughening effects of the cold recycled mixture are achieved through the WER/SBR modifiers. WER and SBR can form a three-dimensional interpenetrating structure, which then cross-links with emulsified asphalt, improving the interfacial bonding strength between emulsified asphalt and the aggregate. Xu et al. [37] employed emulsified asphalt modified with WER for CMA and showed that this modification can improve the early strength of CMA mixtures. Regardless of the WER type and dosage, the emulsified asphalt exhibits strong high-temperature stability and water resistance, although its low-temperature properties are comparatively weaker.

Currently, WER is usually made by dispersing oil-based epoxy resins in water. There are two primary techniques for preparing WER: phase inversion and chemical modification. However, in both methods, epoxy resins must first be synthesized and then dispersed in water, each with its own limitations. In the phase inversion method, controlling the particle size of the dispersed phase is challenging, which results in poor stability of WER [38]. Chemical modification methods involve altering the epoxy resin molecules or breaking epoxy groups, leading to a lower cross-linking density in WER [39].

The main objective of this study is to propose a new method for preparing WER by combining the preparation process of oil-based epoxy resin with the aqueous dispersion process to tackle these challenges. This approach aims to simplify the production process and reduce environmental pollution while detailing its production principles and processes. WER is utilized to enhance emulsified asphalt, ultimately resulting in waterborne epoxy-acrylate resin emulsified asphalt (WEREA). It investigates the fundamental properties of WEREA and its potential to improve CMA mixtures through thorough testing and analysis. Additionally, the study proposes methods for mix design, specimen formation, and curing of WEREA mixtures and compares their key performances with those of HMA.

## 2. Materials and Methods

### 2.1. Preparation of WEREA

**Figure 1:**
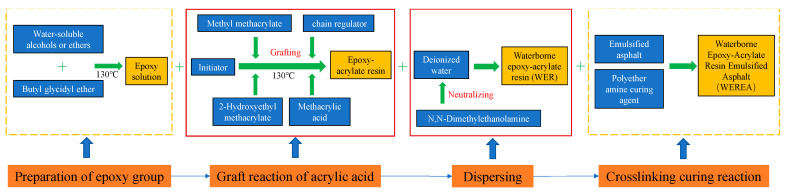
Preparation process of WEREA.

**Figure 2:**
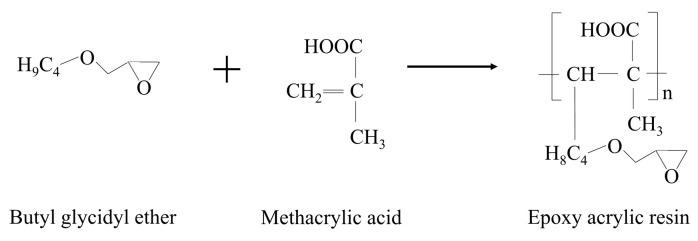
Process of the grafting reaction.

The preparation of WEREA encompasses two key steps: (1) Firstly, a novel WER formulation was developed, involving modifications to the epoxy resin molecules to ensure optimal compatibility and cross-linking with emulsified asphalt; (2) subsequently, the WEREA was prepared by incorporating the WER and curing agent (modified polyether amine) into the emulsified asphalt, ensuring uniform dispersion and adequate interaction. The preparation process can be interpreted with Figure 1 as follows:

The synthesis process of WER involves two distinct steps aimed at integrating epoxy groups onto acrylic resin molecules and subsequently dispersing the resulting epoxy-acrylate resin in water to obtain WER. This integration is achieved through the use of epoxy-reactive diluents, which play a dual role. Firstly, they act as solvents to dilute the acrylic resin, enabling its involvement in the reaction. Secondly, these diluents can directly graft onto acrylic molecules at a later stage. The grafting reaction process is depicted in Figure 2. This synthesis approach streamlines the process, leading to significant reductions in VOC emissions and cost savings.

The primary reaction in the preparation process involves the grafting of epoxy groups onto methacrylic acid [40]. During this reaction, the carbon–carbon double bonds present in methacrylic acid undergo opening, initiating the polymerization of methacrylic acid to generate polyacrylic acid resin. Concurrently, epoxy-reactive diluents are grafted onto the polyacrylic acid resin, leading to the formation of epoxy-acrylate resin. Additionally, the properties of emulsified asphalt, WER, and the curing agent are outlined in Table 1 and Table 2.

In the preparation process, the determination of the ratio between WER and the curing agent is based on considerations of their chemical composition and the number of functional groups they possess. Adjustments to the concentrations of WER and the curing agent are made to approach integer ratios. After experimentation, the optimal ratio between WER and the curing agent is identified as 2:1. This specific ratio is found to produce favorable results under the given testing conditions and objectives of the study.

Table 3 presents various mixtures of emulsified asphalt, WER, and the curing agent, with the parameters denoting mass ratios. The preparation process for WEREA involves blending emulsified asphalt, WER, and the curing agent. The mixing duration is 30 min at a rate of 100 rpm, and the mixing temperature is maintained at 25 °C. The demulsification time is defined as the duration it takes for all the water in WEREA to evaporate, causing it to lose flowability. Table 3 indicates that the mass of WER and the curing agent have no discernible effect on the demulsification time.

### 2.2. Properties of WEREA

#### 2.2.1. FM Tests

Fluorescence microscopy (FM) is widely employed to analyze the microstructure of polymer-modified asphalt [41,42,43]. To investigate the microstructure of WEREA under different proportions of WER, samples of WEREA-0, WEREA-2, WEREA-4, WEREA-6, and WEREA-8 were prepared for observation, both at a magnification of 20× and at a testing temperature of 25 °C. Due to the hardness of the samples, they were placed directly on the slide without using a cover slip and observed using an inverted fluorescence microscope.

#### 2.2.2. FTIR Tests

Fourier Transform infrared spectroscopy (FTIR) is used to identify the chemical functional groups in a substance, which are key to various reactions within the compound [44]. The wavenumber range extends from 400 to 4000 cm^−1^, with 32 scans performed. The FTIR analysis was performed at 25 °C.

#### 2.2.3. DSR Tests

The dynamic shear rheometer (DSR) is a crucial instrument for characterizing the rheological properties of materials. In this study, both temperature and frequency sweep tests were performed using the DSR to assess the performance of WEREA under varying conditions. The temperature sweep tests help evaluate the material’s behavior across a range of temperatures, while frequency sweep tests provide insights into its viscoelastic properties at different frequencies. These analyses are essential for understanding how WEREA performs in practical applications and under different environmental conditions.

In the frequency sweep test, the analysis was conducted at a fixed temperature of 70 °C, with the frequency varying from 0.1 to 100 rad/s. At the same time, the temperature sweep test was carried out by changing the temperature from 46 to 82 °C while keeping the frequency constant at 10 rad/s. These tests provided critical data, including the complex modulus ($G^{*}$) and phase angle (φ). The complex modulus indicates the material’s stiffness, while the phase angle shows its capacity to dissipate energy. Additionally, the rutting factor ($G^{*}/\sin\varphi$) and fatigue factor ($G^{*}\cdot \sin\varphi )$ were assessed from the results. This experimentation also explored how varying WER content affects the rheological properties of WEREA.

#### 2.2.4. MSCR Tests

Domingos and Faxina [45] identified the Multiple Stress Creep Recovery (MSCR) test as a preferred method for evaluating the creep recovery capability of asphalt materials. Evaluation parameters include the creep recovery ratio (R), non-recoverable creep compliance ($J_{nr}$), and relative difference of creep recovery rate ($R_{diff}$). The test protocol follows the specifications of AASHTO T350, and the temperature for testing was controlled at 64 °C.

#### 2.2.5. LAS Tests

Based on the Viscoelastic Continuum Damage (VECD) theory, Linear Amplitude Sweep (LAS) tests are recommended for evaluating the fatigue characteristics of asphalt materials under repeated loading conditions [46]. According to AASHTO TP 101-12-UL test specifications, the LAS test comprises frequency sweep and amplitude sweep segments. First, a frequency sweep is carried out at a 0.1% strain level across a frequency range of 0.2 to 30 Hz to determine the relationship between the storage modulus ($G^{\prime }$) and frequency. After a 2 min relaxation period, an amplitude sweep is conducted at 10 Hz, with strain varying from 0.1% to 30%. The test is performed at a temperature of 25 °C.

#### 2.2.6. Direct Tension Tests

**Figure 3:**
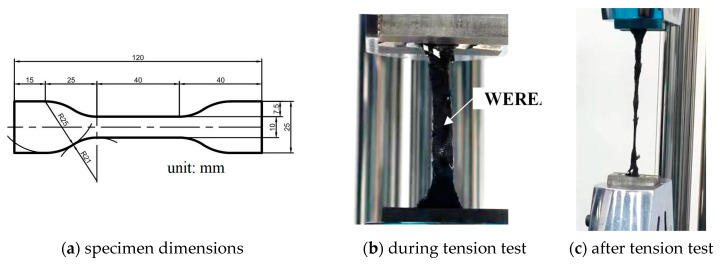
Direct tension tests for WEREA.

A direct tension test was performed on dumbbell-shaped WEREA specimens at 25 °C to evaluate how WER affects their tensile properties. The specimens, as depicted in Figure 3, have dimensions of 120 mm in length, 25 mm in end width, and 5 mm in thickness. A loading rate of 500 mm/min was used, and the strength was measured by the ratio of the peak tensile load to the cross-sectional area of the central section of the specimen. The tension rate can be calculated using Equation (1).

(1) $$TR=\frac{\Delta L}{40}=\frac{L-40}{40}$$

where TR stands for tensile rate; L corresponds to the final length of the middle part; ΔL is the increased length of the middle part.

## 3. Results

### 3.1. Results from FM Tests

**Figure 4:**
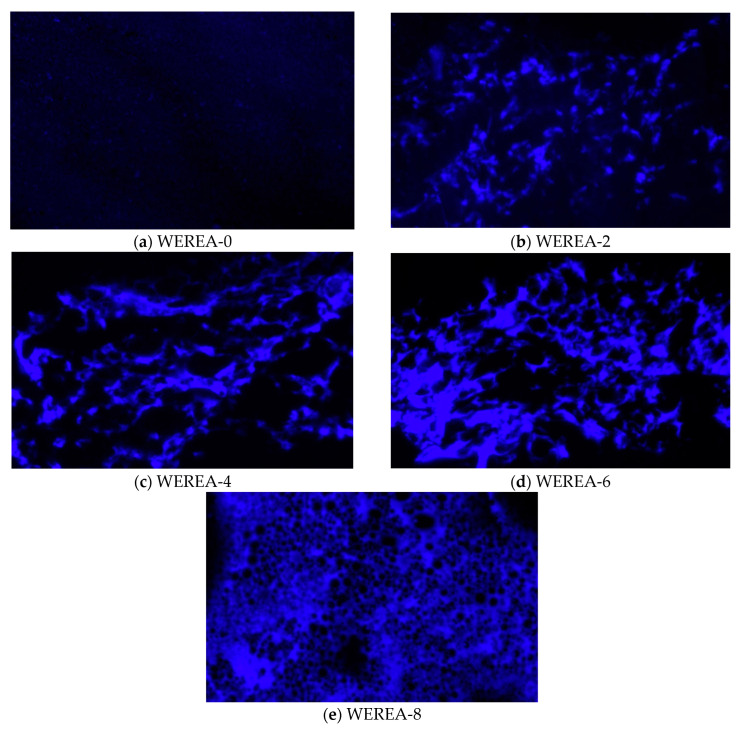
Results of fluorescence microscope tests (with a magnification of 20×).

Figure 4 shows the results of fluorescence microscope tests, which could provide more accurate information about the microstructure of the material and thus be utilized to analyze the fusion state and microscopic characteristics of the polymers. It can be observed that when WER is not added, the image of WEREA-0 is predominantly black, indicating that the asphalt does not exhibit fluorescent properties and there are no bright spots in the fluorescence microscope image. The few bright spots seen in the image may be due to a small amount of SBR rubber or other organic compounds added to the emulsified asphalt. Figure 4b–e show bright regions, demonstrating that WER has fluorescent properties. The distribution of WER in Figure 4b appears relatively random and has a small volume, suggesting that the skeletal structure formed by WER is not yet fully developed. In Figure 4c,d, the volume and density of the WER skeletal structure gradually increase, and a honeycomb-like network structure begins to appear, though the voids are still relatively large. In Figure 4e, the WER skeletal structure is fully formed, with a dense and evenly distributed structure, with asphalt distributed within its voids. This indicates that the skeletal structure of WEREA is formed by WER, and as the WER content increases, the structure becomes more compact.

### 3.2. Results from FTIR Tests

**Figure 5:**
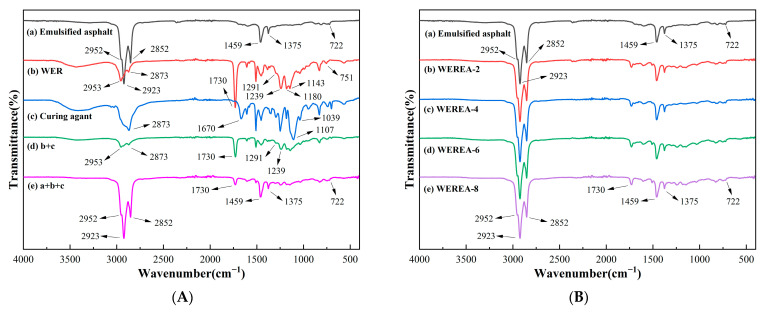
Results from FTIR tests. (**A**) Infrared spectra of various components of WEREA. (**B**) Infrared spectra of WEREA with different WER contents.

FTIR spectroscopy tests are employed in the study to identify and characterize the composition of the composite materials. A change in the characteristic pattern of absorption bands could clearly reflect the change in the composition of the synthetic polymers. The results from FTIR tests are illustrated in Figure 5. From Figure 5A, it can be observed that all sample groups exhibit absorption peaks around 2953 cm^−1^, 2952 cm^−1^, 2923 cm^−1^, 2873 cm^−1^, and 2852 cm^−1^. The peaks at 2953 cm^−1^ and 2873 cm^−1^ are due to the stretching vibrations of methyl (–CH_3_) groups, while the peak at 2952 cm^−1^ corresponds to the asymmetric stretching vibration of methyl groups. The peak at 2923 cm^−1^ is attributed to the asymmetric stretching vibration of methylene (–CH_2_) groups, and the peak at 2852 cm^−1^ is due to the symmetric stretching vibration of methylene groups. These groups reflect the common characteristics of the organic materials in the samples.

In addition to the aforementioned positions, emulsified asphalt also shows absorption peaks at 1459 cm^−1^, 1375 cm^−1^, and 722 cm^−1^. The peak at 1459 cm^−1^ is due to the asymmetric bending vibrations of methyl or methylene groups, while the peak at 1375 cm^−1^ results from the symmetric bending vibrations of methyl groups. The peak at 722 cm^−1^ is attributed to the rocking vibrations of methylene groups. These characteristic absorption peaks indicate that the asphalt molecules are primarily composed of long-chain alkane molecules, which aligns with existing research findings.

WER shows characteristic absorption peaks at 1730 cm^−1^, 1291 cm^−1^, 1239 cm^−1^, 1180 cm^−1^, and 1143 cm^−1^. The peaks at 1291 cm^−1^ and 1239 cm^−1^ are due to the asymmetric stretching vibrations of C–O–C bonds, while the peaks at 1180 cm^−1^ and 1143 cm^−1^ are due to the symmetric stretching vibrations of C–O–C bonds. This suggests that WER contains epoxy groups grafted onto acrylic resin. The peak at 1730 cm^−1^ is due to the stretching vibrations of the C=O bond in carboxyl groups, which are present in the acrylic resin main chain of the WER molecule.

The curing agent shows absorption peaks at 1670 cm^−1^, 1107 cm^−1^, and 1039 cm^−1^. The peak at 1670 cm^−1^ is due to the bending vibrations of the N–H bond in the amino group, which is a characteristic absorption peak for amine curing agents. The peaks at 1107 cm^−1^ and 1039 cm^−1^ are due to the stretching vibrations of ether bonds (C–O–C), indicating that the curing agent is a polyether amine.

From curve (d), it can be seen that, compared to curves (b) and (c), the characteristic peaks of epoxy groups at 1180 cm^−1^ and 1143 cm^−1^, as well as the amino group peak at 1670 cm^−1^, have largely disappeared, indicating that the reaction between WER and the curing agent is nearly complete. Curve (e) shows an additional peak at 1730 cm^−1^ compared to curve (a), which is attributed to the unreacted C=O bonds in the WER molecule. The rest of the spectrum is similar to curve (a), suggesting that the blended system of WER and emulsified asphalt retains the characteristics of emulsified asphalt without generating new absorption peaks. The characteristic peaks of WER and the curing agent are almost absent in WERA, and the infrared spectrum of WERA is not simply a superposition of individual components, indicating a homogeneous system. This analysis suggests good compatibility between the components and indicates that the system is a simple physical blend without the formation of new substances. In addition, the infrared spectra of WEREA with different WER contents are shown in Figure 5B. It can be seen that the infrared spectra of WEREA with different WER contents are largely consistent, indicating that regardless of the WER content, there is good compatibility with emulsified asphalt.

### 3.3. Results from DSR Tests

**Figure 6:**
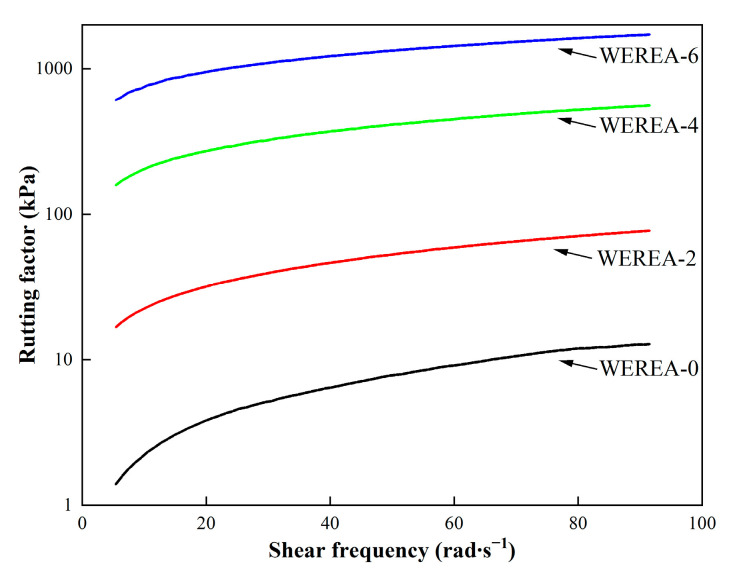
Rutting factors under different shear frequencies.

The residue of WEREA-8 demonstrates remarkable resistance to softening, even when exposed to temperatures as high as 120 °C. It is worth noting that none of the rheology tests have been conducted on WEREA-8. In the realm of asphalt binder, the rutting factor ($G^{*}/\sin\varphi$) is a critical parameter for assessing high-temperature stability, with higher values of $G^{*}/\sin\varphi$ indicating enhanced rutting resistance. The rutting factors, as depicted in Figure 6, are obtained from frequency sweep tests carried out at 70 °C. It is observed that as shear frequency increases, there is a corresponding increase in rutting factors. Moreover, with the increase in WER content (from WEREA-0 to WEREA-6), the rutting factor shows a significant improvement.

**Figure 7:**
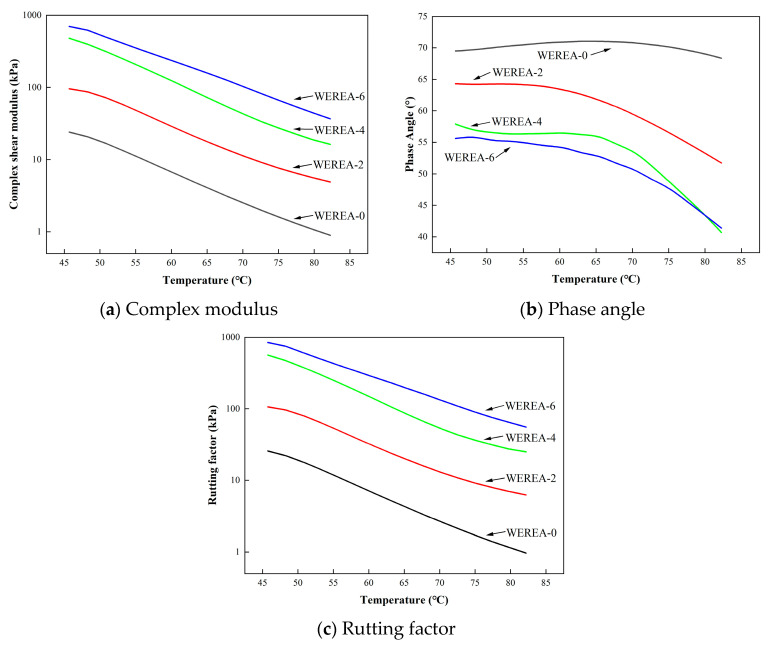
Results of temperature sweep test.

The results of the temperature sweep test can be observed in Figure 7. The temperature sweep test results show that the complex shear modulus significantly decreases with rising temperature. However, an increase in WER content leads to a notable rise in the complex shear modulus, reflecting improved mechanical performance. The phase angle, in contrast, remains stable with temperature increases below 60 °C. However, when the temperature exceeds 60 °C, the phase angle demonstrates a decreasing trend, signifying a decrease in material viscosity due to the elevated temperature. This observation highlights the thermosetting characteristics of epoxy resin, which are fundamentally distinct from the viscoelastic behavior of asphalt binders. Notably, for WEREA-0, the phase angles are larger compared to other samples, indicating a higher viscosity for these two types of WEREA. At the same temperature, an increase in WER content leads to a notable decrease in the phase angle, indicating a higher proportion of storage modulus and a lower proportion of viscous modulus. As a result of the incorporation of WER, WEREA exhibits an augmented rutting factor with increasing content.

### 3.4. Results from MSCR Tests

**Figure 8:**
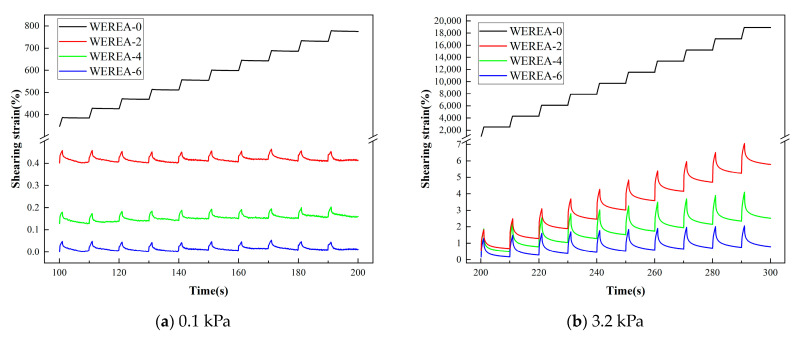
Shear strain–time curve under different stresses.

Figure 8 depicts the shear strain versus time curves of specimens under two stress levels at 64 °C. It is evident from the graph that, regardless of the stress level applied, WEREA undergoes shear deformation under loading, with some of the deformation recovering upon stress unloading. The unrecoverable deformation accumulates with each cycle, increasing as the number of cycles grows. At stress levels of 0.1 kPa and 3.2 kPa, the shear strain of WEREA decreases with higher WER content, suggesting that adding WER enhances the hardness of the emulsified asphalt and improves its resistance to deformation.

**Figure 9:**
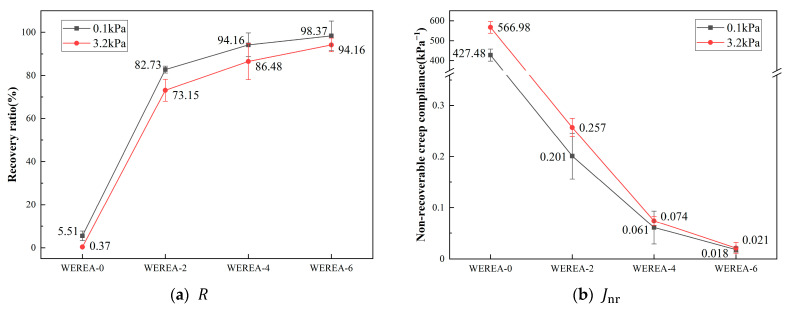
Results of the MSCR test.

The resistance to rutting of WEREA is evaluated using two indicators, R and, $J_{nr}$ which are generally inversely proportional, as illustrated in Figure 9. It can be observed from the graph that, under both stress levels, R increases gradually with an increase in WER content. In particular, under stress levels of 0.1 kPa and 3.2 kPa, WEREA-6 exhibits a 1685% and 25,348% increase in R compared to WEREA-0, respectively. The behavior of $J_{nr}$ is opposite to that of R, decreasing by over 99% under stress levels of 0.1 kPa and 3.2 kPa. The combined variation of R and $J_{nr}$ indicates that WER significantly enhances the elasticity and hardness of emulsified asphalt, transitioning it from a viscoelastic material to an elastic material, thereby improving its deformation resistance, resistance to rutting, and high-temperature performance.

**Figure 10:**
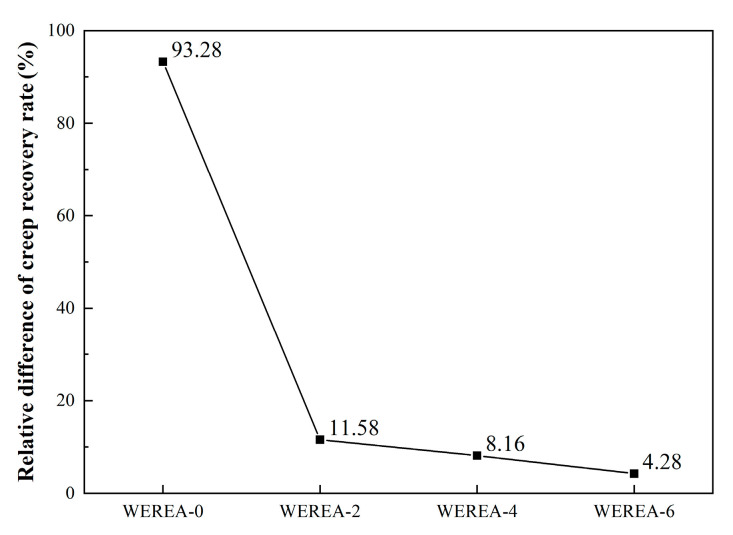
The results of $R_{diff}$ calculations.

In addition to R and $J_{nr}$, $R_{diff}$ is another crucial indicator for evaluating asphalt materials. $R_{diff}$ reflects the sensitivity of WEREA to stress changes, with smaller $R_{diff}$ values indicating lower sensitivity of R to stress changes, and thus better high-temperature stability. The results of $R_{diff}$ calculations are presented in Figure 10. It is evident from the graph that, with an increase in WER content, $R_{diff}$ continuously decreases. This suggests that under stress levels of 0.1 kPa and 3.2 kPa, the relative difference in creep recovery rate of WEREA becomes smaller, indicating a reduced sensitivity of R to stress changes. From WEREA-0 to WEREA-6, $R_{diff}$ decreases by 95.4%, demonstrating that WER enhances the deformation resistance of emulsified asphalt.

### 3.5. Results from LAS Tests

#### 3.5.1. Amplitude Sweep

**Figure 11:**
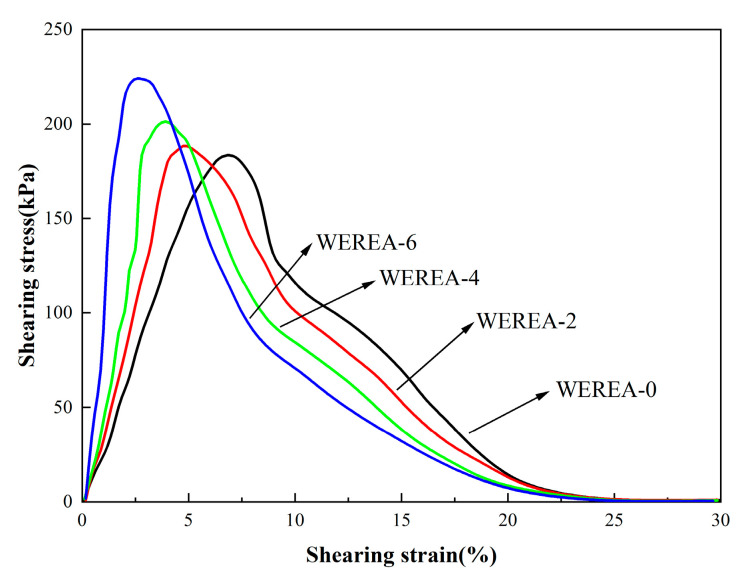
Results of the amplitude sweep.

After performing the frequency sweep, an amplitude sweep is conducted on the specimen with shear strain varying from 0.1% to 30%. This amplitude sweep provides the shear stress–shear strain curve, which is crucial for analyzing the specimen’s resistance to deformation. The results of the amplitude sweep are illustrated in Figure 11.

It is evident that the shear stress rises with increasing shear strain. However, after reaching a peak value, the shear stress begins to decrease as the shear strain continues to increase, signaling the failure of the specimen. Comparing different samples from WEREA-0 to WEREA-6, it can be observed that the shear stress at failure progressively increases while the strain at failure decreases. This indicates that with higher WER content, the emulsified asphalt’s resistance to deformation improves, but its ductility diminishes.

#### 3.5.2. Fatigue Life

**Figure 12:**
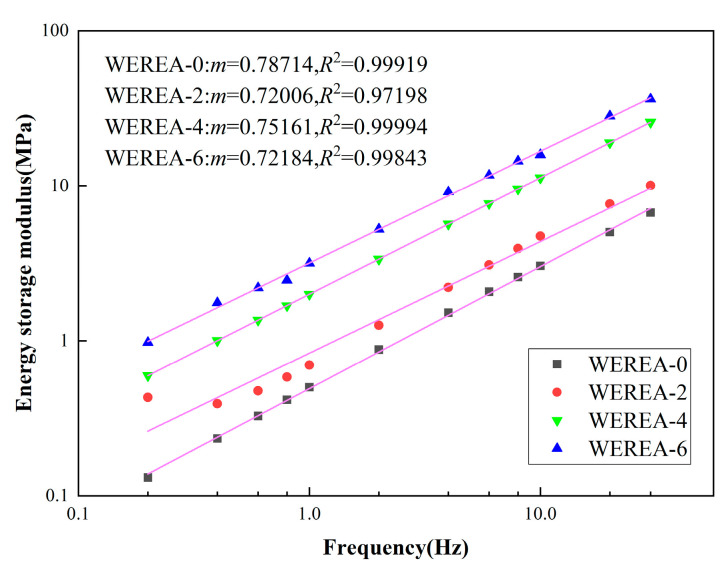
Results of the frequency sweep.

To calculate the fatigue life, the results of the frequency sweep need to be fitted, and the parameter α is calculated. The fitting results are shown in Figure 12, and the value of α is presented in Table 4.

**Figure 13:**
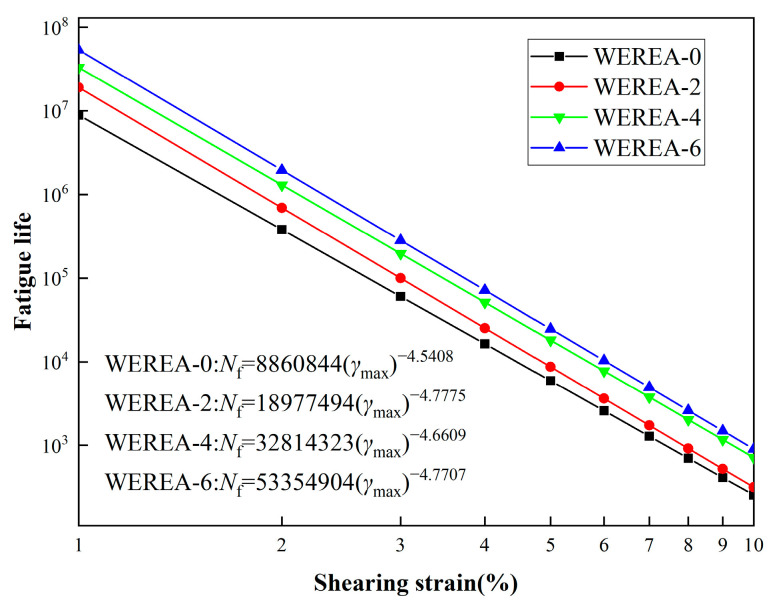
Relationship between fatigue life and shear strain.

Table 4 lists the additional parameters needed for calculating fatigue life. Figure 13 illustrates the relationship between WEREA fatigue life and strain for maximum expected strains ranging from 1% to 10% across different WER contents. The equations of the fatigue life can also be obtained in the figure.

From Figure 13, it is observed that the fatigue life of WEREA decreases progressively with increasing shear strain. For a given shear strain, the fatigue life improves as the WER content increases, indicating that WER contributes to enhancing the fatigue life of emulsified asphalt. Based on the trend shown in the graph, it can be estimated that the fatigue life of WEREA-8 might increase by an order of magnitude compared to WEREA-0. These findings are consistent with the results from the amplitude sweep, both demonstrating that higher WER content improves the deformation resistance of emulsified asphalt.

**Figure 14:**
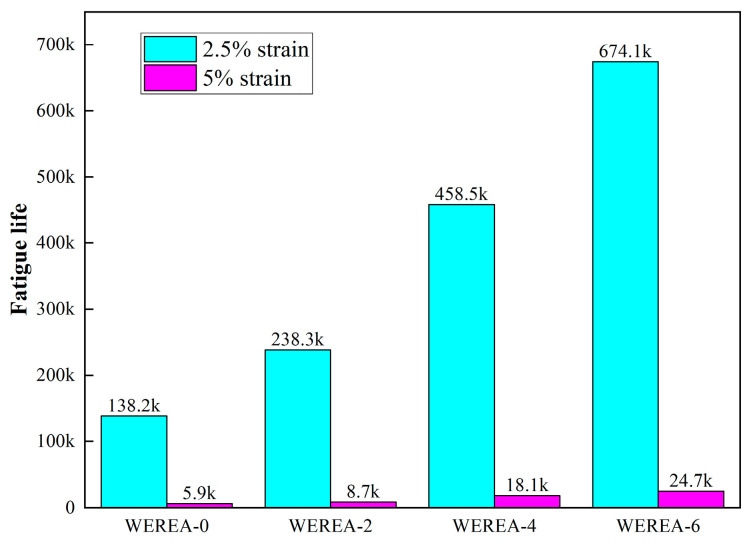
Fatigue life of WEREA at 2.5% and 5% strain.

Regarding the maximum expected strain ($\gamma_{max}$), research by Ashish et al. suggests that it should be 5% for low-intensity pavements and 2.5% for high-intensity pavements [47]. Figure 14 shows the fatigue life of WEREA at maximum expected strains of 2.5% and 5%. The graph reveals that WEREA-6 exhibits a 387% increase in fatigue life at 2.5% strain and a 316% increase at 5% strain compared to WEREA-0. This demonstrates that WER significantly improves the fatigue life of emulsified asphalt at both strain levels.

### 3.6. Results from Direct Tension Tests

**Figure 15:**
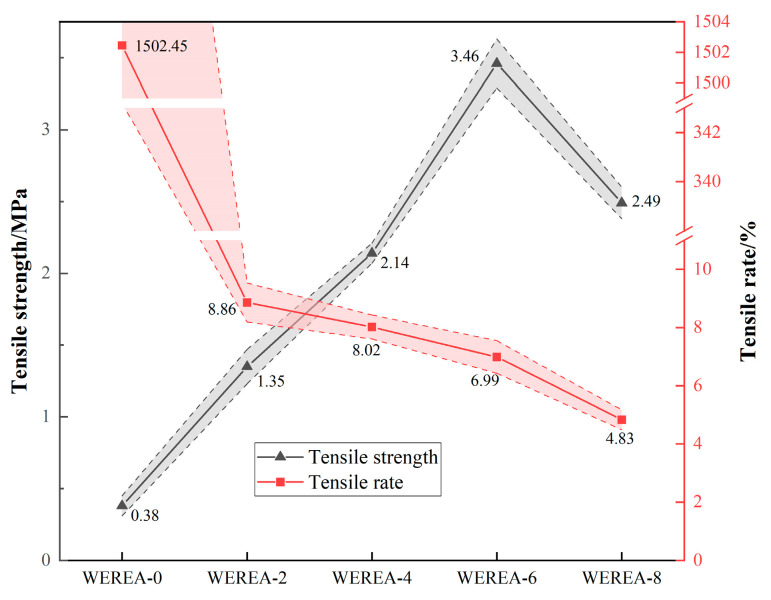
Results of direct tension test.

Figure 15 illustrates the tensile outcomes of WEREA fabricated with varying amounts of WER. In the absence of WER, WEREA-0 displayed minimal tensile strength but exhibited significant elongation at breaking, reaching up to 1500%. This suggests that emulsified asphalt demonstrated commendable deformation capacity but lacked desirable tensile properties in the absence of WER modification. Upon the incorporation of WER, the tensile rate at break experienced a sharp decline, but the tensile strength underwent a more substantial improvement. This implies that WER significantly enhances the tensile strength of emulsified asphalt.

In the transition from WEREA-0 to WEREA-6, the tensile strength surged by 811%, accompanied by a substantial reduction in deformation capacity, reaching up to 99.5%. The pinnacle of tensile strength was reached at WEREA-6, beyond which it declined. This decline could be attributed to excessive doping of WER, rendering WEREA more brittle and less resilient to deformation, consequently diminishing its ability to withstand external loads.

## 4. Discussion

CMA has been widely applied in practical engineering. Based on the rheological and mechanical properties of WEREA, the WEREA mixture exhibits better performance for intermediate layers. However, the mix design for CMA is still in the exploratory stage. According to current research findings and Chinese standards [48,49], this paper conducted a mix design for the WEREA mixture.

### 4.1. Fabrication of WEREA Mixture

#### 4.1.1. Materials and Process of Mix Design

**Figure 16:**
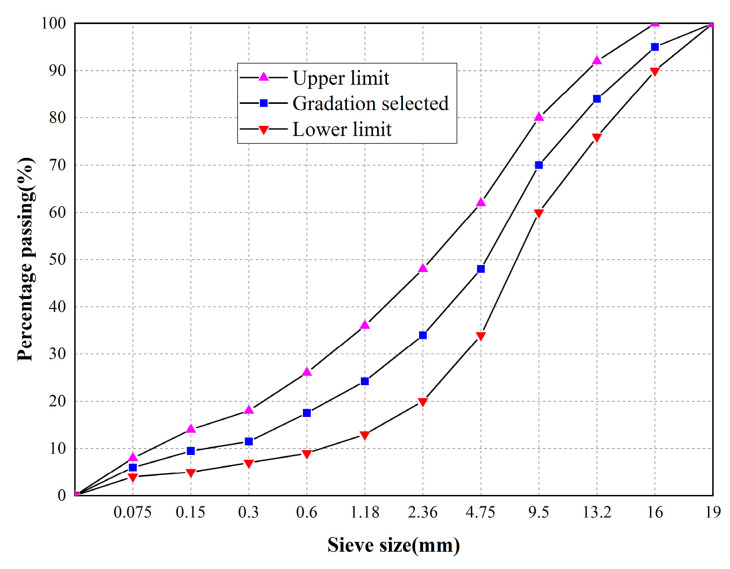
Gradation of AC-16.

The WEREA mixture consists of WEREA, aggregate, mineral powder, cement, and external water. According to the previous study on the fatigue and mechanical properties of WEREA, it is observed that the best fatigue and mechanical performance is achieved when the ratio of emulsified asphalt to WER + curing agent is 1:0.6. Although other ratios may be more suitable for WEREA mixtures, this ratio is chosen in this study for illustrative mix design. The WEREA mixture uses limestone aggregate graded as AC-16, and its gradation is shown in Figure 16.

The amount of mineral powder significantly influences the performance of the mixture. Insufficient amounts may fail to fill the voids between aggregates, leading to excessive void content. Conversely, excessive amounts can reduce asphalt adhesion, resulting in decreased strength. Therefore, the mineral powder is used in the quantity required for AC-16 gradation, which is 6%.

When the cement content is too high, the mixture tends to become dry and hard, making it difficult to mix. On the other hand, too little cement prevents adequate dispersion of water in the mixture, hindering specimen molding and subsequent testing. As per the Chinese standard [49], the cement quantity is equal to the amount replaced by mineral powder. Therefore, the total amount of cement and mineral powder is 6%, with cement substituting part of the mineral powder.

**Figure 17:**
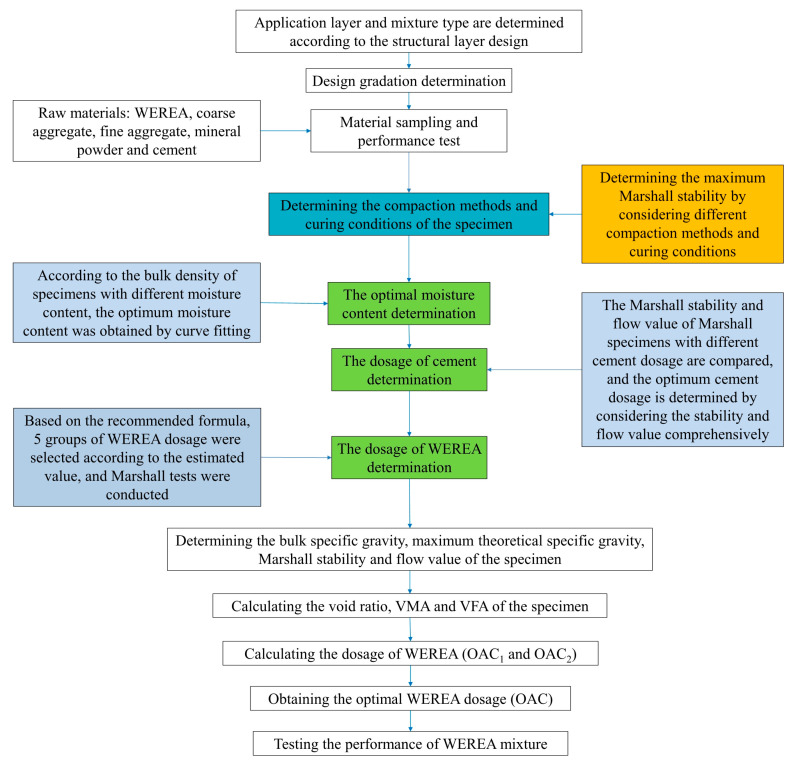
The process of the mix design for the WEREA mixture.

The main process of the mix design is illustrated in Figure 17.

#### 4.1.2. Compaction Methods and Curing Conditions for Specimens

(1) Determination of Marshall Compaction Methods

**Figure 18:**
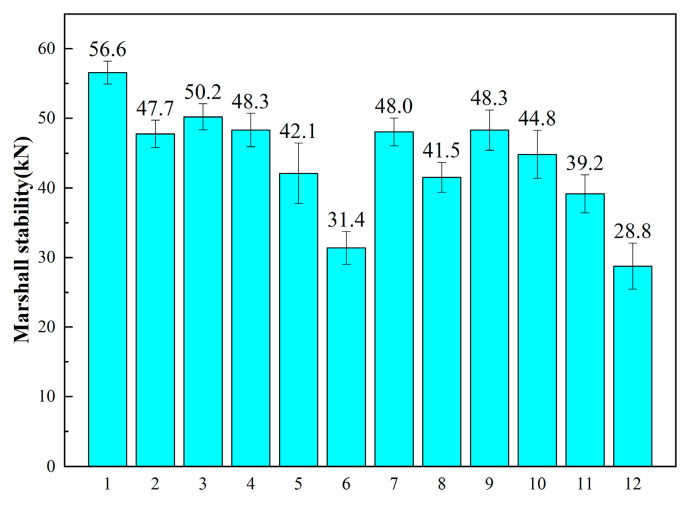
Results of different compaction methods.

Different compaction methods are employed for Marshall specimens to investigate their impact on stability and determine the optimal compaction method. WEREA content is set at 9.5%, cement content at 1.5%, curing temperature at 60 °C, and total curing time at 48 h. The cement initial setting time is 142 min, and various compaction methods are detailed in Table 5, with corresponding results illustrated in Figure 18.

It can be observed that using the “50 + 25” compaction method results in lower stability compared to the “25 + 25” under the same conditions. This indicates that excessive initial compaction may expel the emulsion, leading to reduced strength. Additionally, employing a single compaction method yields higher strength than compaction in two stages, suggesting that any subsequent compaction disrupts the formed epoxy resin and cement structures. Specifically, a second compaction after 24 h damages the structure significantly, causing a substantial strength decrease. Therefore, for the mix design, a single compaction method with 50 compactions on each side is recommended.

(2) Determination of the Curing Temperature and Curing Time

**Figure 19:**
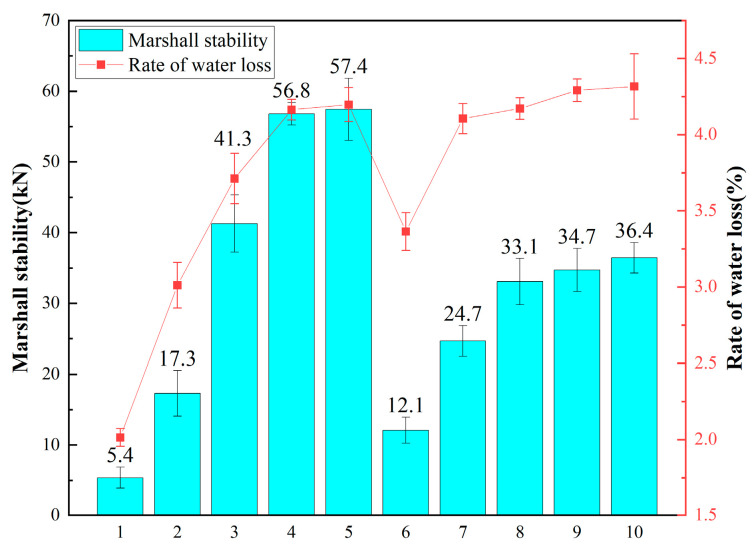
Results of different curing conditions.

Two curing temperatures for CMA, 110 °C and 60 °C, are considered [49]. Existing research suggests that high curing temperatures lead to rapid water loss, increasing void content and decreasing mixture strength. Conversely, low curing temperatures prolong curing time. This study explores the water loss rate and strength of the mixture over time at both temperatures to determine the optimal curing conditions. Emulsion and cement contents, compaction method, and curing conditions are detailed in Table 6, with results shown in Figure 19.

Figure 19 demonstrates that at 60 °C and 110 °C, mixture strength gradually increases over time, reaching its maximum around 48 h. Although strength development is slower at 60 °C, the 6 h and 12 h strengths are smaller than those at 110 °C, indicating that higher temperature accelerates water evaporation and strength development. However, the final strength after curing at 60 °C surpasses that at 110 °C. This discrepancy may be attributed to excessive water evaporation at higher temperatures, resulting in larger internal voids and lower final strength. Furthermore, water loss rates indicate that at 60 °C, the maximum water loss occurs at 48 h, while at 110 °C, it occurs at 12 h. This underscores that higher temperatures expedite water evaporation but compromise final strength. Therefore, the chosen curing conditions are 60 °C for 48 h.

#### 4.1.3. Determination of Optimal Moisture Content

**Figure 20:**
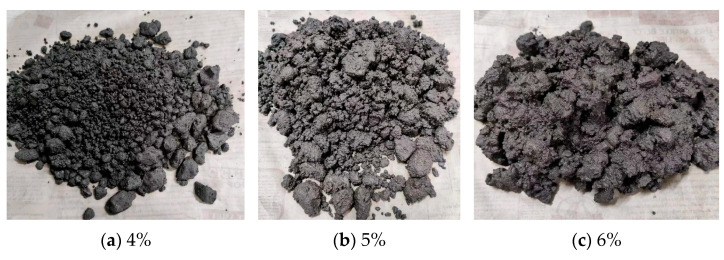
The states of the mixture at different moisture contents.

The optimal moisture content is determined through compaction tests, where the specimen’s moisture content affects its dry density. The maximum dry density corresponds to the optimal moisture content. Recommended emulsion and cement contents for compaction tests are 3.5% and 1.5%, respectively. The states of the mixture at different moisture contents are depicted in Figure 20. It illustrates that at 4% moisture content, the mixture is relatively dry, with minimal free-flowing emulsion and some clumping, indicating poor uniformity. As the moisture content increases, the mixture’s uniformity and flowability improve. At 5% moisture content, the mixture exhibits good uniformity. Continuing to 6% moisture content, the mixture becomes soft and prone to collapse, showing good uniformity.

**Figure 21:**
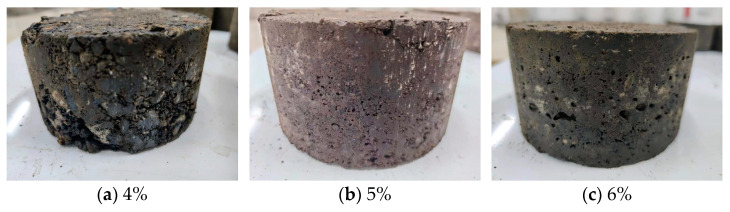
Marshall specimens with different moisture contents after curing.

Figure 21 presents Marshall specimens with different moisture contents after curing. At 4% moisture content, the specimen exhibits poor adhesion, with aggregates separating due to insufficient filling, resulting in large surface voids. At 6% moisture content, the specimen’s surface is smooth but shows more voids due to water evaporation. At 5% moisture content, the Marshall specimen surface is smooth, with fewer voids.

**Figure 22:**
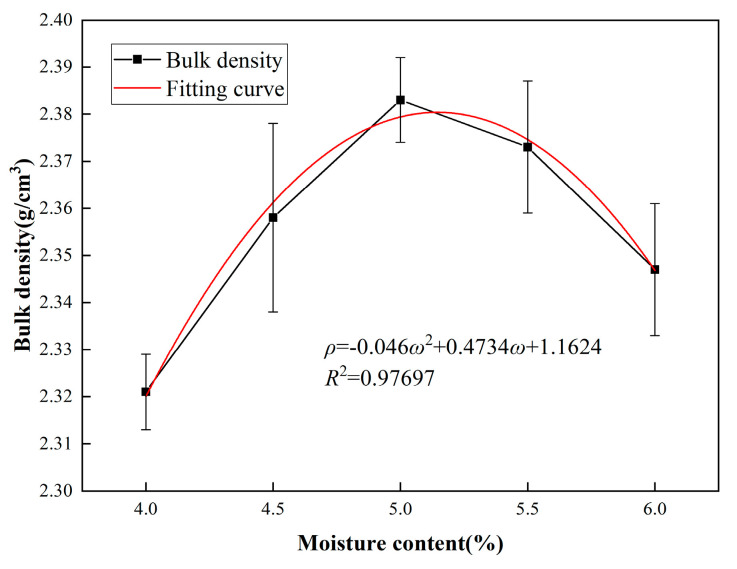
Bulk density and fitting curve of different moisture contents.

The results of bulk density tests are shown in Figure 22. It indicates a consistent trend between bulk volume density and uniformity. Both increase with moisture content up to around 5%, then decrease. This suggests that at optimal uniformity, the maximum bulk volume density is achieved, corresponding to the minimum void content. Fitting a quadratic function to the bulk volume density curve reveals the symmetrical axis, indicating the optimal moisture content is 5.14%.

#### 4.1.4. Determination of Cement Dosage

**Figure 23:**
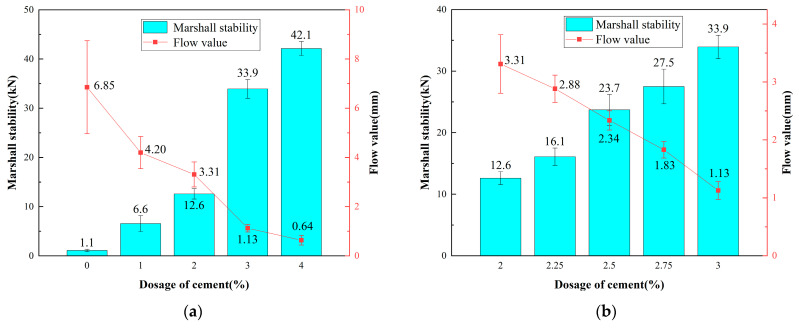
(**a**,**b**) Marshall stability and flow value at different cement contents.

According to the Chinese specifications [49], the method used in this study is the cement replacement method with mineral powder. By replacing part of the cement with mineral powder, the optimal cement content is explored, and the evaluation criteria are Marshall stability and flow value. The replacement ratio ranges from 0% to 4%. The WEREA content is 9.5%, and the moisture content is 5.14%, which means the external water content is 0.105%. The experimental results of Marshall stability and flow value at different cement contents are shown in Figure 23.

It is clear that cement content significantly affects the Marshall stability and flow value of the specimens. Stability increases with higher cement content; for instance, at 0% cement, stability is just 1.1 kN, likely due to insufficient strength from the mineral powder and lack of hydration reactions. At 4% cement, Marshall stability rises to 42.1 kN, showing that cement enhances specimen strength effectively. Conversely, the flow value decreases with increasing cement content. At 0% cement, the flow value is 6.85 mm, indicating a loose structure and higher displacement upon failure. At 4% cement, the flow value drops to 0.64 mm, reflecting improved strength but reduced ductility. To achieve a balance between stability and flow value, a final cement content of 2.5% is chosen.

#### 4.1.5. Determination of WEREA Content

The WEREA content of the WEREA mixture is determined according to Equation (2).

(2) P=0.06A+0.12B+0.2C

where P stands for the percentage by mass of WEREA in the total aggregate; A stands for the percentage by mass of aggregate particles larger than 2.36 mm in the total aggregate; B stands for the percentage by mass of aggregate particles between 0.075 mm and 2.36 mm in the total aggregate; C stands for the percentage by mass of aggregate particles smaller than 0.075 mm in the total aggregate.

**Figure 24:**
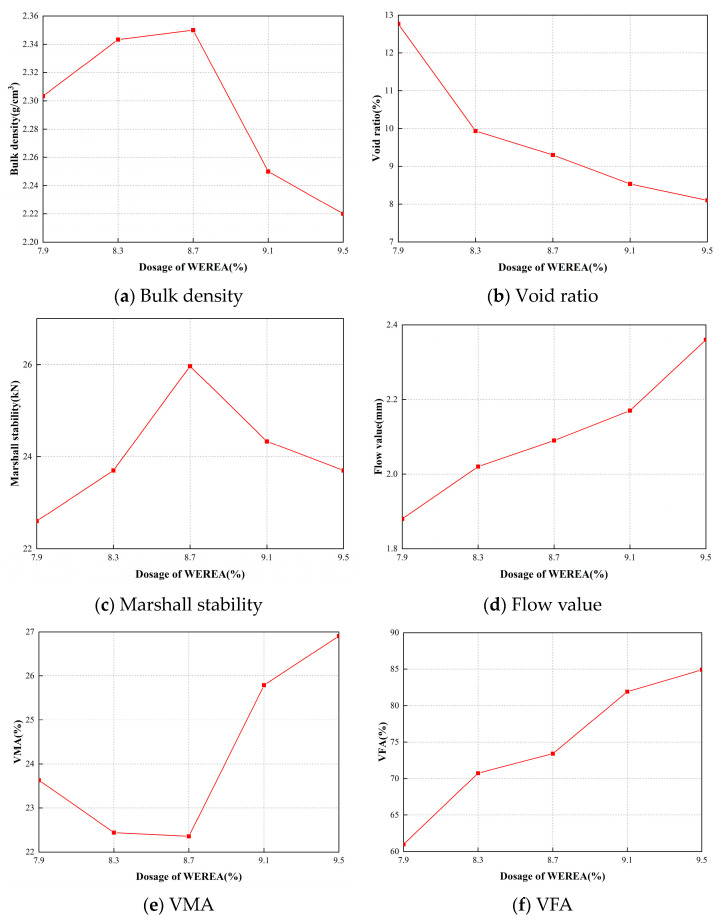
Results of Marshall tests.

Using Equation (2), the emulsion content is calculated to be 8.52%. Therefore, five WEREA contents—7.9%, 8.3%, 8.7%, 9.1%, and 9.5%—are selected for Marshall tests. However, when changing the WEREA content, the external water content must be adjusted to maintain the same moisture content of the mixture. Based on this, the external water content for varying WEREA levels is calculated and detailed in Table 7. The cement content is set at 2.5%, with 50 compactions on each side during the compaction process and curing at 60 °C for 48 h. The results of the Marshall test are illustrated in Figure 24.

According to the Chinese standard [49], the optimal asphalt content (OAC) is obtained by averaging ${\mathrm{OAC}}_{1}$ and ${\mathrm{OAC}}_{2}$. The calculation methods for ${\mathrm{OAC}}_{1}$ and ${\mathrm{OAC}}_{2}$ are shown in Equation (3) and Equation (4), respectively:

(3) $${\mathrm{OAC}}_{1}=\frac{a_{1}+a_{2}+a_{3}+a_{4}}{4}$$

where $a_{1}$ stands for the dosage of WEREA, which corresponds to the maximum of Marshall stability; $a_{2}$ stands for the dosage of WEREA, which corresponds to the maximum bulk density; $a_{3}$ stands for the dosage of WEREA, which corresponds to the target of void ratio; $a_{4}$ stands for the dosage of WEREA, which corresponds to the midpoint of VFA.

(4) $${\mathrm{OAC}}_{2}=\frac{{\mathrm{OAC}}_{\min}+{\mathrm{OAC}}_{\max}}{2}$$

where ${\mathrm{OAC}}_{\min}$ stands for the dosage of WEREA where all indicators (excluding VMA) meet the lower limit of technical standards; ${\mathrm{OAC}}_{\max}$ stands for the dosage of WEREA where all indicators (excluding VMA) meet the upper limit of technical standards.

Calculating ${\mathrm{OAC}}_{1}$ using Equation (3), where $a_{1}$ and $a_{2}$ are both 8.7%. For $a_{3}$, considering the specified void ratio for emulsified asphalt mixtures in the Chinese standard [49], generally ranging from 8% to 13%; the midpoint of 10.5% void ratio is taken as the target void ratio, corresponding to $a_{3}$ being 8.2%. For a nominal maximum particle size of 19 mm mixture, the required asphalt saturation is 60% to 75%, with a midpoint of 67.5%, and $a_{4}$ is interpolated accordingly to be 8.1%. Thus, ${\mathrm{OAC}}_{1}=\left(8.7\%+8.7\%+8.2\%+8.1\%\right)/4=8.425\%$.

**Figure 25:**
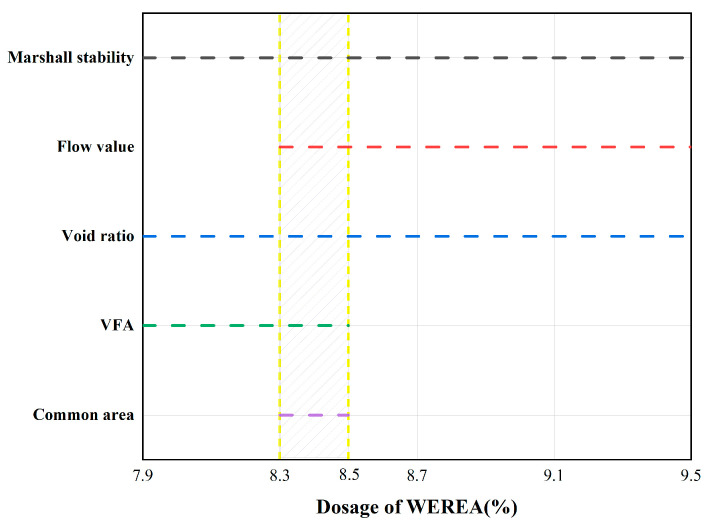
The dosage of WEREA that all indicators meet the requirements.

Calculating ${\mathrm{OAC}}_{2}$ using Equation (4). The dosage of WEREA that all indicators meet the requirements of the standard is represented by the shaded part in Figure 25; thus, ${\mathrm{OAC}}_{\min}$ and ${\mathrm{OAC}}_{\max}$ can be obtained. The shaded area in the figure represents the emulsion content meeting the specification requirements. From this, ${\mathrm{OAC}}_{\min}$ is determined to be 8.3%, and ${\mathrm{OAC}}_{\max}$ is 8.5%. Therefore, ${\mathrm{OAC}}_{2}=\left(8.3\%+8.5\%\right)/2=8.4\%$.

Under normal conditions, the optimal emulsion content is the average of ${\mathrm{OAC}}_{1}$ and ${\mathrm{OAC}}_{2}$, i.e., OAC $=\left(8.425\%+8.4\%\right)/2=8.4125\%$, where 8.4% was chosen as the optimal emulsion content of the mixture.

### 4.2. Performance of WEREA Mixture in Pavement Application

After the mix design of the WEREA mixture, this section investigates the pavement performance of the WEREA mixture, including high-temperature performance, low-temperature fracture performance, and water stability, compared with the AC-16 mixture. All WEREA mixtures in this section adopt the optimal mix design obtained from Section 4.1, with a WEREA content of 8.4%, cement content of 2.5%, and moisture content of 5.14%.

#### 4.2.1. High-Temperature Stability

**Figure 26:**
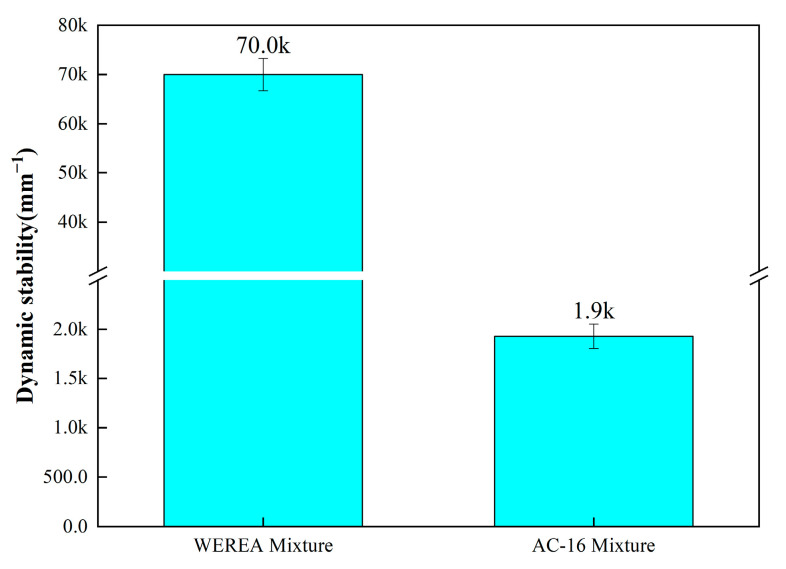
Results of rutting tests.

The high-temperature stability of the WEREA mixture is assessed using rutting tests. Standard rutting specimens are prepared, and tests are performed at 60 °C with a wheel frequency of 42 cycles per minute for 1 h. Dynamic stability, the evaluation criterion, is directly measured by the rutting test instrument. Results are illustrated in Figure 26.

As depicted in Figure 26, the dynamic stability of the WEREA mixture is markedly higher compared to the AC-16 mixture, demonstrating superior high-temperature stability. This enhanced performance is likely due to the improved high-temperature characteristics of the solidified WER product, which exhibits greater stiffness and deformation resistance at elevated temperatures. This change positively affects the thermoplastic properties of the asphalt, making it less prone to deformation under repeated vehicle loading and thereby enhancing resistance to rutting. Additionally, the results of DSR tests also reflect this characteristic of WEREA. At high temperatures, WEREA exhibits a higher rutting factor, indicating better high-temperature stability.

#### 4.2.2. Fracture Performance

At low temperatures (−10 °C), asphalt exhibits brittleness, while at moderate temperatures (25 °C), it demonstrates pronounced viscoelastic behavior. Similarly, asphalt concrete shows brittle cracking at low temperatures, whereas, at moderate temperatures, a noticeable plastic zone appears at the crack tip, making it inappropriate to consider the cracking as purely brittle. To assess crack resistance at both low and moderate temperatures, different evaluation methods are required.

**Figure 27:**
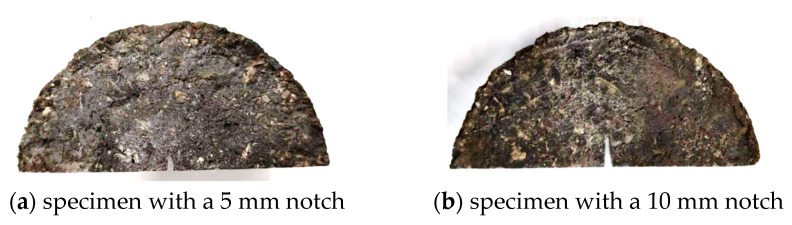
Semi-circular specimens with notches of different depths.

The Semi-Circular Bend (SCB) test can be employed to assess the crack resistance of asphalt concrete [50]. In this test, a semi-circular specimen is cut and loaded until failure, generating a load–displacement curve used to evaluate crack resistance. Semi-circular specimens, with a diameter of 101.6 mm and a thickness of 63.5 mm, are prepared from standard Marshall specimens by slicing them in half. A pre-cut notch, 1.5 mm wide, is made at the specimen’s center to mimic an initial crack. Notch depths of 5 mm and 10 mm are used, as shown in Figure 27. For low-temperature SCB tests, a 5 mm notch is employed, while the moderate-temperature test utilizes both 5 mm and 10 mm notches.

(1) Low-Temperature Fracture Performance

The crack resistance of asphalt concrete at low temperatures is assessed using two parameters: the stress intensity factor $K_{I}$ and the fracture energy $G_{F}$. As the crack propagates, $K_{I}$ increase, and when they reach a specific critical value $K_{\mathrm{IC}}$, the crack will experience unstable extension. This critical value $K_{\mathrm{IC}}$ is referred to as the fracture toughness, which is a crucial parameter for characterizing the crack resistance of asphalt concrete.

For asphalt concrete:

(5) $$K_{\mathrm{IC}}=\frac{P_{\max}}{2Rt}\sqrt{\pi a}Y_{I}$$

where R stands for the radius of the semi-circular specimen, mm; t stands for the thickness of the semi-circular specimen, mm; $P_{max}$ stands for the peak load, kN; a stands for the depth of the notch, mm; $Y_{I}$ stands for the shape factor, which is influenced by the ratio of the span distance between the supports to the diameter of the semi-circular specimen.

**Figure 28:**
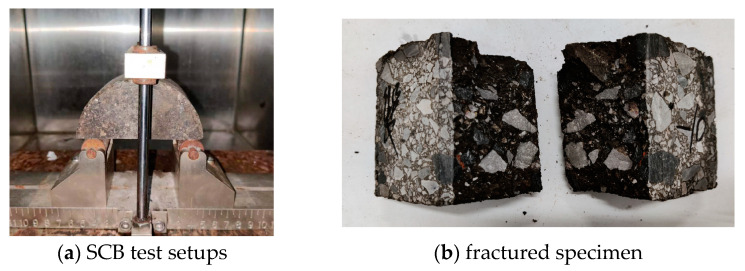
SCB test setups and results.

The SCB test is performed with a span-to-diameter ratio of 0.8, as illustrated in Figure 28. The test temperature is 0 °C. Lim and Johnston provided methods for calculating values at different span-to-diameter ratios [51], with the calculation method for a ratio of 0.8 given by Equation (6).

(6) $$Y_{I,0.8}=4.782-1.219\left(\frac{a}{R}\right)+0.063\exp\left(7.045\left(\frac{a}{R}\right)\right)$$

From Equation (6), the shape factor $Y_{I}$ is calculated to be 4.7875.

**Figure 29:**
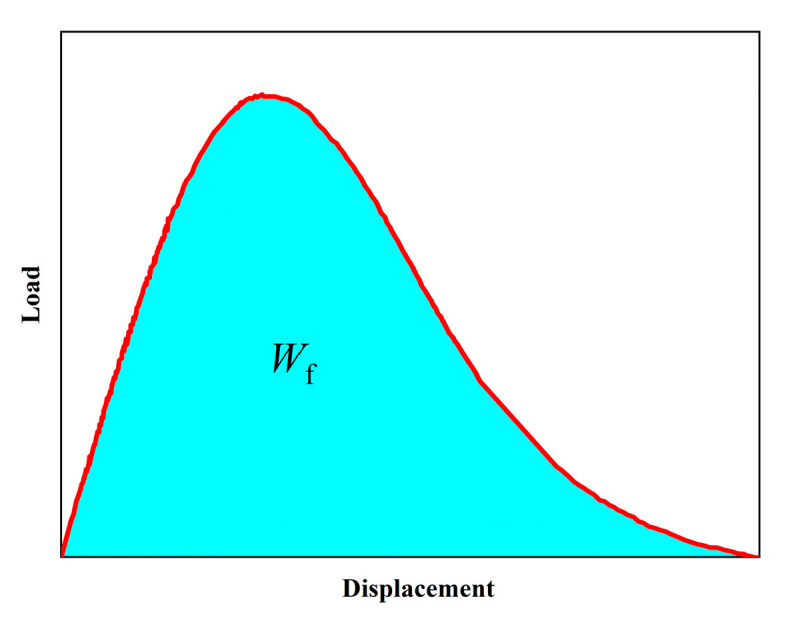
The load–displacement curve of a semi-circular specimen.

The fracture process of asphalt concrete involves energy absorption and dissipation, and its crack resistance can be assessed through energy conservation and transformation. Fracture energy measures the energy released per unit area as a crack extends, representing the work done during crack extension. It is relatively easy to calculate and measure. Figure 29 shows a typical load–displacement curve of a semi-circular specimen. The fracture energy of asphalt concrete can be approximated using Equation (7).

(7) $$G_{F}=\frac{W_{f}}{A_{\mathrm{lig}}}$$

where $W_{f}$ stands for the total energy released during the fracture process; $A_{lig}$ stands for the area over which the crack extends.

**Figure 30:**
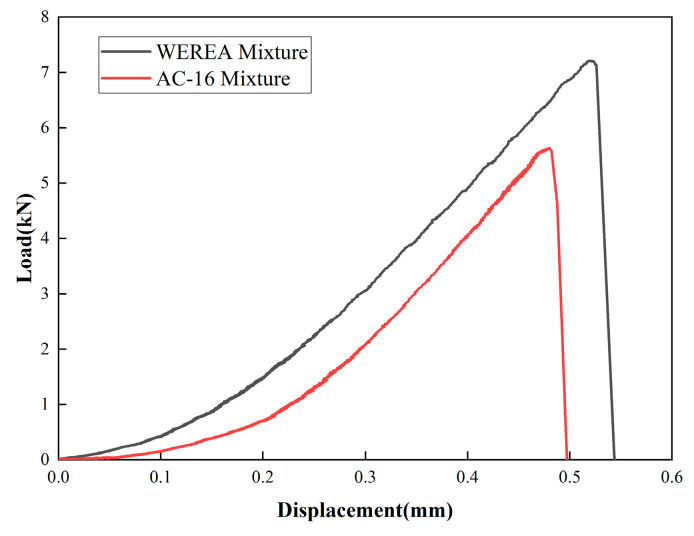
Results of the low-temperature SCB test.

Figure 30 presents the results of the low-temperature SCB test. It can be observed that both the WEREA mixture and AC-16 mixtures show a linear relationship between load and displacement, followed by a rapid decline after reaching the peak load, indicating brittle fracture at low temperatures.

**Figure 31:**
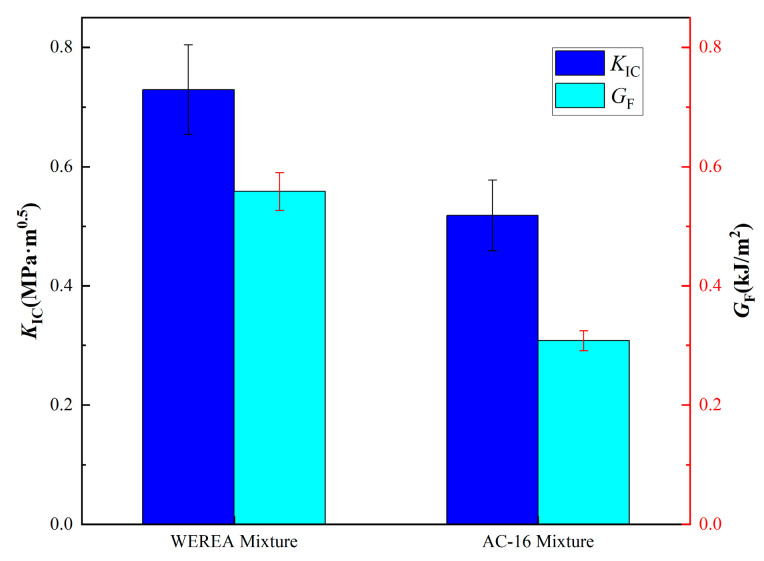
Calculated $K_{\mathrm{IC}}$ and $G_{F}$.

The calculated $K_{\mathrm{IC}}$ and $G_{F}$ for the WEREA mixture and AC-16 mixtures at low temperatures are shown in Figure 31. The WEREA mixture exhibits a 40.7% higher $K_{\mathrm{IC}}$ than AC-16, suggesting its ability to withstand higher loads during low-temperature fracture. The $G_{F}$ of the WEREA mixture is 81.2% higher than that of AC-16, indicating greater energy absorption per unit area during fracture. This may be attributed to the higher stiffness of the solidified WER, providing greater load-bearing capacity and energy absorption compared to the AC-16 mixture at low temperatures.

(2) Moderate-Temperature Fracture Performance

When asphalt concrete fractures at moderate temperatures, there is a significant plastic zone at the crack tip, and its size cannot be ignored. Therefore, the stress intensity factor based on linear elastic theory cannot be used for evaluation. In such cases, the J-integral is generally used to assess the crack resistance of asphalt concrete under nonlinear conditions.

For asphalt concrete, the calculation method for the J-integral is given by Equation (8).

(8) $$J=\left(\frac{U_{1}}{t_{1}}-\frac{U_{2}}{t_{2}}\right)\times \frac{1}{a_{2}-a_{1}}$$

where $U_{1}$ stands for the area under the load–displacement curve of specimen 1 from the origin to the peak load, J; $U_{2}$ stands for the area under the load–displacement curve of specimen 2 from the origin to the peak load, J; $t_{1}$ stands for the thickness of specimen 1, mm; $t_{2}$ stands for the thickness of specimen 2, mm; $a_{1}$ stands for the depth of the notch in specimen 1, mm; $a_{2}$ stands for the depth of the notch in specimen 2, mm, and $a_{1}\neq a_{2}$.

**Figure 32:**
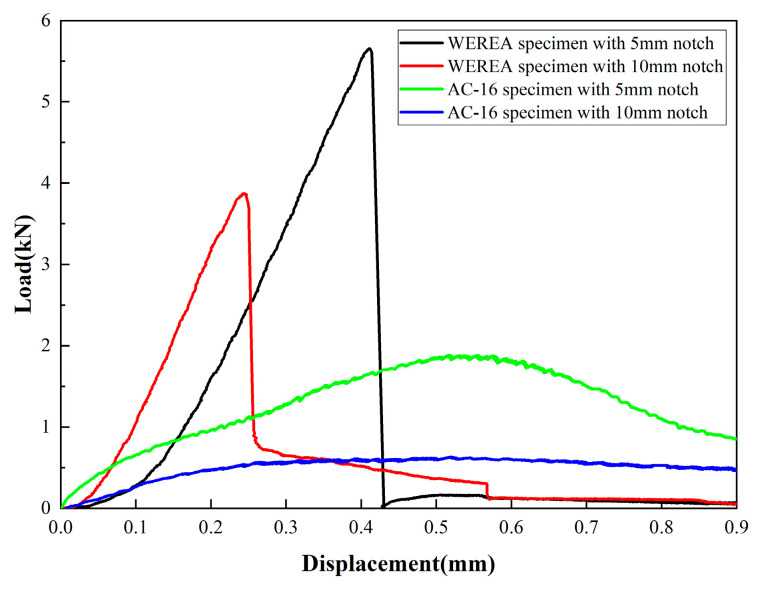
Results of the moderate-temperature SCB test.

In the moderate-temperature SCB tests conducted at 25 °C, as shown in Figure 32, the load–displacement curve for the WEREA mixture is similar to that observed at low temperatures, displaying a linear relationship between load and displacement. After reaching the peak load, the load drops rapidly, indicating that the WEREA mixture exhibits higher stiffness and undergoes brittle fracture at moderate temperatures. The load–displacement curve for the AC-16 mixture, on the other hand, shows plastic characteristics, with a nonlinear relationship between load and displacement before reaching the peak load and a gradual decrease in load with displacement afterward, reflecting the viscoelastic nature of asphalt at moderate temperatures.

**Figure 33:**
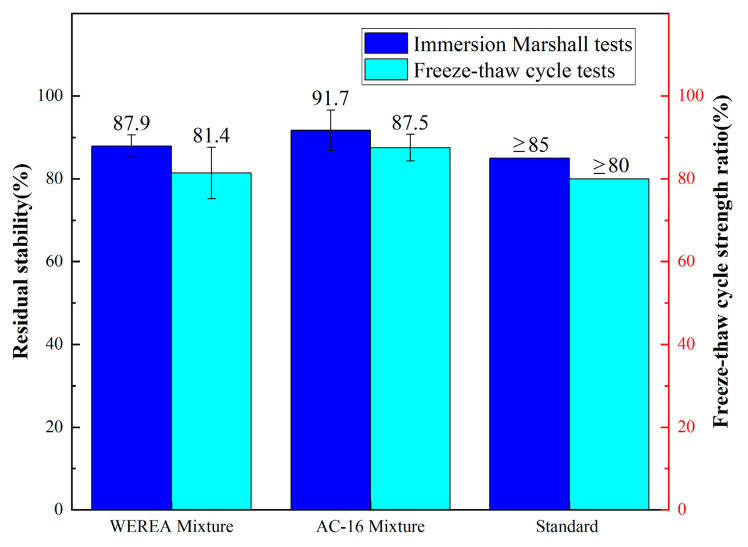
Results from the immersion Marshall tests and freeze–thaw cycle tests.

The J-integral for both WEREA and AC-16 mixtures, calculated using Equation (8), are shown in Figure 33. It can be seen that at moderate temperatures, the J-integral for the WEREA mixture is 43.3% higher than that for the AC-16 mixture, indicating that more energy is required for the fracture of the WEREA mixture at moderate temperatures. This suggests that the WEREA mixture enhances the moderate temperature crack resistance of asphalt concrete. Although the fracture modes of the two mixtures differ at moderate temperatures, using the J-integral for crack resistance evaluation may be inappropriate. However, the load–displacement curves reflect that the WEREA mixture has higher stiffness at moderate temperatures and is less prone to deformation.

#### 4.2.3. Water Stability

After curing, WEREA mixtures tend to develop voids due to internal moisture evaporation. In practical applications, water infiltration through voids in asphalt mixtures can weaken the adhesion between asphalt and aggregate, causing loosening and stripping of the pavement. Common methods to evaluate water stability include the immersion Marshall residual stability (MS_0_) test and the freeze–thaw cycle strength ratio (TSR) test.

Results from the immersion Marshall tests and freeze–thaw cycle tests, as depicted in Figure 33, indicate that the water stability and freeze–thaw cycle strength of the WEREA mixture are both lower compared to the AC-16 mixture. This suggests that the WEREA mixture is less resistant to water-induced damage and freeze–thaw effects than the AC-16 mixture. This could be attributed to the increased porosity in the WEREA mixture due to the evaporation of water, leading to higher void content and increased susceptibility to water damage, thereby reducing water stability compared to the AC-16 mixture. A possible solution to improve the water stability of the WEREA mixture is to add an appropriate amount of calcium hydroxide (Ca(OH)_2_) during the mixing process. Ca(OH)_2_ can react with the water in the emulsified asphalt, thereby reducing the moisture content of the mixture and achieving a decrease in porosity. Additionally, the reaction between Ca(OH)_2_ and water is exothermic, which can accelerate the curing of the epoxy resin and potentially improve the early strength of the mixture. Regardless, the water stability of the WEREA mixture already meets the specified requirements, so this paper does not conduct further tests aimed at enhancing water stability.

## 5. Conclusions

This paper introduced a novel preparation method for a type of WER. This WER was then utilized to modify emulsified asphalt, which subsequently served as a binder for the fabrication of the WEREA mixture. The rheological properties and tensile behavior of the material were determined through rheological and direct tensile tests. The optimal composition of the WEREA mixture was determined through mix design and compared with the AC-16 mixture for road performance. A thorough examination of the results yields the following conclusions:

1. WER demonstrates good compatibility with emulsified asphalt, and regardless of the WER content, it consistently acts as a continuous phase in the WEREA, enveloping the asphalt within the skeletal structure formed after the reaction with the curing agent. As the WER content increases, a denser, more uniform structure with fewer voids is formed, enhancing the strength of the cured structure;
2. With rising temperature, the complex shear modulus of WEREA decreases; when the temperature exceeds 60 °C, the material’s viscosity reduces, and the phase angle shows a decreasing trend. Incorporating WER into emulsified asphalt transitions it from a viscous material to an elastic one, improving its resistance to rutting; as WER content increases, the complex shear modulus and rutting factor rise, while the phase angle decreases. Creep test results indicate that WER significantly enhances the elasticity and hardness of emulsified asphalt while decreasing the sensitivity of creep recovery rate to stress changes. Fatigue test results show that the fatigue life of WEREA-6 under 2.5% strain is 387% longer than that of WEREA-0 and 316% longer under 5% strain;
3. When WER content is 60%, the optimal compaction method for the AC-16 designed WEREA mixture is double-sided compaction 50 times, with the best curing conditions at 60 °C for 48 h, optimal moisture content at 5.14%, optimal cement dosage at 2.5%, and optimal WEREA dosage at 8.4%. WEREA improves the high-temperature stability of the mixture, with dynamic stability counts far exceeding those of the AC-16 mixture. When failure occurs, the WEREA mixture can withstand greater loads and absorb more energy per unit area during crack propagation. At low temperatures, the stress intensity factor of the WEREA mixture is 40.7% higher than that of the AC-16 mixture, and the fracture energy is 81.2% higher. At medium temperatures, the J-integral of the WEREA mixture is 43.3% higher than that of the AC-16 mixture. The water immersion residual stability and freeze–thaw cycle strength of the WEREA mixture decreased by 4.14% and 6.97%, respectively, compared to the AC-16 mixture but still met the specified requirements;
4. Overall, the incorporation of WER into emulsified asphalt offers significant improvements in the rheological, tensile, and fatigue properties of the resulting WEREA mixture. Moreover, the WEREA demonstrated better high-temperature stability and low-temperature performance than AC-16 but slightly lower water stability. This suggests that WEREA could be a promising alternative to traditional asphalt mixtures, particularly in applications where high-temperature stability, low-temperature performance, and durability are critical;
5. For the future application of the material, due to the desirable cohesive and adhesive properties of WEREA, it can be explored for use in producing cold recycled asphalt mixtures. This approach can better leverage the material’s performance advantages and enhance the utilization rate and efficiency of recycled asphalt pavement (RAP), achieving energy-saving and environmentally friendly results. In addition to designing the WEREA mixture according to AC-16 gradation for use in the middle layer of asphalt pavements, consideration can also be given to using a gradation with a smaller nominal maximum aggregate size. By optimizing the material proportions, WEREA mixtures could be applied in the surface layer or ultra-thin wearing course.

## Figures and Tables

**Table 1:** Basic properties of emulsified asphalt.

Materials	Items	Unit	Requirement	Test Results
Emulsified asphalt	Demulsification speed	/	slow	slow setting
	Solid content	%	≥50	50.2
Residue of emulsified asphalt	Ductility (10 °C)	mm	≥450	1300
	Penetration (100 g, 25 °C, 5 s)	0.1 mm	≥40	92.4
	Softening point	°C	>50	66.2
Storage stability	1 day	%	≤1	0.21
	5 days	%	≤5	1.34

**Table 2:** Basic properties of WER and the curing agent.

Materials	Appearance	Density (g/mL)	Solid Content (%)	pH	Engler Viscosity	Epoxy Equivalent (g/mol)	Total Amine Value (mg KOH/g)
WER	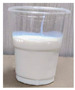	1.21	50.83	7.07	15.7	1485.86	/
Curing agent	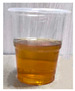	1.04	24.16	/	23.7	/	73.7

**Table 3:** Mix proportion of WEREA.

Groups	Emulsified Asphalt	WER	Curing Agent	WER + Curing Agent	Demulsification Time
WEREA-0	1	0	0	0	45 min
WEREA-2		0.133	0.067	0.2	
WEREA-4		0.267	0.133	0.4	
WEREA-6		0.4	0.2	0.6	
WEREA-8		0.533	0.267	0.8	

**Table 4:** Parameters of fatigue life.

	α	$C_{0}$	$C_{1}$	$C_{2}$	$A_{35}$
WEREA-0	2.2704	5.3863	0.5041	0.3128	8,860,844
WEREA-2	2.3887	6.9842	0.5332	0.2993	18,977,494
WEREA-4	2.3305	8.5685	0.5706	0.3025	32,814,323
WEREA-6	2.3853	10.1012	0.6034	0.3139	53,354,904

**Table 5:** Compaction methods of WEREA mixture.

Groups	Number of First Compaction	Number of Second Compaction	Interval between Two Compactions (h)
1	25	25	0
2			1.5
3			3
4			6
5			12
6			24
7	50	25	0
8			1.5
9			3
10			6
11			12
12			24

**Table 6:** Curing conditions of WEREA mixture.

Groups	Curing Temperature (°C)	Curing Time (h)
1	60	6
2		12
3		24
4		48
5		72
6	110	6
7		12
8		24
9		48
10		72

**Table 7:** Different external water content for different WEREA content.

WEREA Content (%)	Moisture Content (%)	External Water Content (%)
7.9	5.14	0.953
8.3		0.741
8.7		0.529
9.1		0.317
9.5		0.105

## Data Availability

The original contributions presented in the study are included in the article, further inquiries can be directed to the corresponding author.
